# Sinomenine activates astrocytic dopamine D2 receptors and alleviates neuroinflammatory injury via the CRYAB/STAT3 pathway after ischemic stroke in mice

**DOI:** 10.1186/s12974-016-0739-8

**Published:** 2016-10-10

**Authors:** Jing Qiu, Zhongjun Yan, Kai Tao, Yansong Li, Yuqian Li, Jingchen Li, Yushu Dong, Dayun Feng, Huisheng Chen

**Affiliations:** 1 Department of Neurology, The General Hospital of Shenyang Military Region, Shenyang, Liaoning 110016 People’s Republic of China; 2 Department of Neurosurgery, Tangdu Hospital, The Fourth Military Medical University, Xi’an, Shaanxi 710038 People’s Republic of China; 3 Department of Neurology, The 463rd Hospital of PLA, Shenyang, Liaoning 110042 People’s Republic of China; 4 Department of Neurosurgery, The General Hospital of Shenyang Military Region, Shenyang, Liaoning 110016 People’s Republic of China

**Keywords:** Ischemic stroke, Neuroinflammation, Astrocyte, Dopamine D2 receptor (DRD2), Sinomenine

## Abstract

**Background:**

Astrocyte-mediated neuroinflammation plays a critical role in ischemic stroke-induced secondary cerebral injury. Previous studies have suggested that the dopamine D2 receptor (DRD2) acts as a key target in regulating the neuroinflammatory response. However, the underlying molecular mechanisms are still unknown, and effective DRD2 agonists are lacking. In the present study, we examined the anti-inflammatory and neuroprotective effects of sinomenine (Sino), a monomeric compound with potential immunoregulatory properties in nervous system.

**Methods:**

TTC staining, apoptosis assay, evaluation of brain edema, and neurological assessment were performed in the middle cerebral artery occlusion (MCAO) mouse model. Primary astrocytes exposed to oxygen glucose deprivation (OGD) were used in the in vitro experiments. Quantitative PCR was applied to assess the levels of inflammatory cytokines. Multi-labeling immunofluorescence, Western blot, co-immunoprecipitation, and electrophoretic mobility shift assay (EMSA) were also used to investigate the molecular mechanisms underlying the Sino-mediated anti-inflammatory effects in vivo and in vitro.

**Results:**

Sino remarkably attenuated the cerebral infarction and neuronal apoptosis, reduced the levels of inflammatory cytokines, and alleviated neurological deficiency in MCAO mice. Sino significantly inhibited astrocytic activation and STAT3 phosphorylation as well as increased DRD2 and αB-crystallin (CRYAB) expression after MCAO. In vitro, Sino blocked OGD-induced activation of STAT3 and generation of pro-inflammatory cytokines in primary astrocytes, and these effects were significantly abolished by either DRD2 or CRYAB knockdown. Additionally, Sino induced up-regulation and nuclear translocation of CRYAB in astrocytes and enhanced the interaction between CRYAB and STAT3, which further inhibited the activation and DNA-binding activity of STAT3.

**Conclusions:**

Our study demonstrates that Sino activates astrocytic DRD2 and thereby suppresses neuroinflammation via the CRYAB/STAT3 pathway, which sheds some light on a promising therapeutic strategy for ischemic stroke.

**Electronic supplementary material:**

The online version of this article (doi:10.1186/s12974-016-0739-8) contains supplementary material, which is available to authorized users.

## Background

Ischemic stroke is one of the leading causes of morbidity and mortality in adults worldwide and accounts for approximately 87 % of all stroke cases [1]. Although rapid reperfusion is a critical treatment strategy for ischemic stroke, it can paradoxically lead to exacerbated cerebral ischemia and reperfusion (IR) injury. The mechanisms of IR injury involve various pathophysiological processes, among which inflammation has been confirmed by increasing evidences [2, 3]. Thus, modulating inflammatory signals may be a potential therapeutic approach for the treatment of ischemic stroke.

Astrocytes are the most numerous glial cells in the brain and play critical roles in maintaining homeostasis in the central nervous system (CNS). They are dynamically involved in the establishment and maintenance of the blood-brain barrier, trophic support of neurons, ionic and metabolic homeostasis, antioxidant defense, and synaptic transmission [4–8]. In addition, astrocytes can also secrete a series of pro-inflammatory and anti-inflammatory cytokines to modify the ambient microenvironment [9–11]. Accumulating evidences indicate that astrocyte-mediated post-ischemic inflammation contributes to the brain injury [12]. Cerebral ischemia activates astrocytes to release various pro-inflammatory cytokines, such as tumor necrosis factor-alpha (TNF-α) and interleukin-1β (IL-1β), which are crucial for the pathological processes of brain ischemic injury [13]. Recently, studies have demonstrated that activation of the astrocytic dopamine D2 receptor (DRD2) suppressed neuroinflammation in intracerebral hemorrhage and 1-methyl-4-phenyl-1,2,3,6-tetrahydropyridine (MPTP)-induced neurotoxicity [14, 15]. Additionally, αB-crystallin (CRYAB), a heat-shock protein, has been shown to possess anti-inflammatory properties and regulate the astrocytic DRD2-mediated anti-inflammatory pathway in both neurodegenerative disorders and cerebral injury models [14, 16, 17]. These studies suggest astrocytes and DRD2 as potential cellular and molecular targets for the experimental and clinical treatment of inflammatory injury in the CNS.

Sinomenine (Sino) is a bioactive alkaloid originally extracted from the Chinese medicinal herb *Sinomenium acutum* [18]. Sino possesses anti-inflammatory and immunoregulatory properties and has long been used for treating rheumatoid arthritis (RA) in China. In addition, Sino protects against acute lung injury induced by lipopolysaccharide (LPS) [19]. Sino was shown to modulate a series of inflammation-related molecules, including nitric oxide, TNF-α, leukotriene C4, and prostaglandin E3, in LPS-treated macrophages in vitro and in vivo [20, 21]. Sino also decreased the expression of TNF-α and IL-1β in adjuvant-induced arthritic rats [22]. Additionally, a study in kidney IR injury has demonstrated its potential anti-inflammatory role through suppressing nuclear factor-*k*B transcriptional activity. However, whether or not Sino exerts protective effect against cerebral ischemic injury and if any the underlying mechanisms are still unknown.

In the present study, we explored the role of Sino and the underlying mechanisms in cerebral ischemic injury. Our results indicated that Sino suppresses the neuroinflammation via targeting the astrocytic DRD2/CRYAB/STAT3 pathway in cerebral ischemic model in vivo and in vitro, which sheds some light on a promising therapeutic strategy for ischemic stroke.

## Methods

### Animal preparation and MCAO modeling

The adult C57BL/6 mice (20~25 g) used in the present study were obtained from the Laboratory Animal Center of the Fourth Military Medical University. All the procedures and ethics guidelines were approved by the Committee for Experimental Animal Use and Care of the Fourth Military Medical University, China. Efforts were made to minimize the number of animals used and their suffering.

The middle cerebral artery occlusion (MCAO) surgery was performed as previously reported [23, 24]. Briefly, the mice were anesthetized with 1.5–2 % isoflurane mixed with oxygen and nitrogen. A silicone-coated 6-0 suture (Covidien, Mansfield, MA) was gently inserted from the exposed external carotid artery stump to the internal carotid artery and wedged into the circle of Willis to obstruct the opening of the middle cerebral artery. The distance from the bifurcation of the internal/external carotid artery to the middle cerebral artery was approximately 9 ± 1.0 mm. The filament was withdrawn after 60 min of the obstruction. The sham operation was identical but did not include the occlusion of the middle cerebral artery.

### Drug administration

Sino was injected intraperitoneally to mice 6 h after MCAO surgery at doses of 10 and 20 mg/kg daily for 3 days. The injection time points were 6, 24, and 48 h post MCAO. The sham group received the same volume of saline intraperitoneally.

### Measurement of cerebral infarction

Seventy-two hours after MCAO, the mice were given an overdose of anesthesia and decapitated. The brains were carefully removed, and 1-mm-thick coronal slices were collected in pre-warmed 2 % triphenyltetrazolium chloride (TTC) (Sigma, St. Louis, MO, USA) in saline for 10 min, followed by 30 min of fixation in 4 % paraformaldehyde in PBS (pH 7.4) [25]. Then, the pictures were analyzed with NIH ImageJ software to determine the infarct volume. Infarct size was expressed as a percentage of hemispheric volume.

### Measurement of brain edema

The brain edema analysis was performed according to a previously reported method [23]. Briefly, the wet brains were weighed and then immediately dried at 95 °C overnight. The brain water content was calculated as ([wet tissue weight − dry tissue weight] / wet tissue weight) × 100 %.

### Neurological injury severity assessment

The severity of neurological injury was assessed according to a four-tiered grading system and a 21-point scoring system named the Garcia test, as reported previously [26, 27]. The trained investigators were blinded to the control groups and drug-treatment groups in the test.

### Primary astrocyte culture and transfection

Astrocytes were prepared from the cortex of C57BL/6 mice at P0, as described previously [14]. Briefly, the neonatal cortical tissues were dissociated with trypsin, and cells were plated at a density of 5 × 10^7^ cells per 75 cm^2^ flask (Thermo Scientific) in DMEM/Ham’s F12 medium containing 10 % fetal bovine serum (FBS). After 24 h, the culture medium was changed to complete medium (DMEM/F12 medium with 10 % FBS) and was subsequently changed twice per week. Cultures were shaken at 200 rpm/min for 12–18 h to remove other types of neuronal cells after 10 days. When the cells reached 70 % of confluence, they were transfected with small interfering RNA (siRNA) for 72 h via Lipofectamine (RNAi MAX Transfection Reagent, Thermo Scientific). DRD2 siRNA and CRYAB siRNA were purchased from Life Technologies. The sequences of the scramble control siRNA were as follows: sense: r(UUCUCCGAACGUGUCACGU) dTdT; and antisense: r(ACGUGACACGU-UCGGAGAA) dTdT.

### Oxygen glucose deprivation (OGD) management and treatment

The procedures were performed in accordance with a previously described protocol [28]. After purification and siRNA transfection as described above, primary astrocytes were cultured with deoxygenated DMEM without glucose and FBS (Gibco, CA, USA) in an incubator (Thermo Scientific) with premixed gas (1 % O_2_, 94 % N_2_, 5 % CO_2_) for 5 h. Then, the cells were given normal DMEM (Gibco, CA, USA) containing 10 % FBS and placed in a CO_2_ incubator (95 % air and 5 % CO_2_) for 24 h. Cells in the control group were cultured with normal DMEM and 10 % FBS for the same time. After OGD induction, 0.01, 0.1, 1, 10 mM Sino was immediately used to treat astrocytes for 24 h.

### Immunofluorescence and TUNEL staining

Histological assessment was performed on a fixed frozen coronal section (25 μm) with a cryostat as described previously [29]. Fresh antibody dilution buffer (0.01 M PBS containing 3 % normal goat serum and 0.3 % Triton X-100) was used for immunofluorescence staining. Sections were incubated at 4 °C overnight with diluted primary antibodies as follows: mouse anti-GFAP (1:400, Cell Signaling Technology), mouse anti-NeuN (1:1000, Abcam), mouse anti-Iba1 (1:200, Abcam), goat anti-DRD2 (1:200, Abcam), and rabbit anti-p-STAT3 (Tyr705) (1:200, Cell Signaling Technology). The following secondary antibodies were purchased from Abcam: donkey anti-mouse IgG (Alexa Fluor 488/594), donkey anti-goat IgG (Alexa Fluor 488/647), and donkey anti-rabbit IgG (Alexa Fluor 594). Images were obtained using a confocal laser microscope system (FV1000, Olympus, Tokyo, Japan). Cells were also fixed with 4 % paraformaldehyde for 10 min at room temperature. The additional primary antibody was anti-CRYAB (1:100, Abcam). The protocol for cell culture staining was the same as that used for the brain sections.

Terminal deoxynucleotidyl transferase-mediated dUTP nick-end labeling (TUNEL) staining was performed according to the instructions provided with a commercial TUNEL staining kit (QIA39, Merck, Germany). Photos were captured with a confocal microscope, and the TUNEL-positive cells in the cerebral ischemic penumbra were counted manually. TUNEL staining analysis was performed on five slices containing cortical ischemic penumbra per mouse (*n* = 5) and five mice per group (*n* = 5).

### Western blot

Tissues containing ischemic penumbra were harvested for Western blotting at 24, 48, 72 h after MCAO following a standard protocol (Molecular Clone, Edition II). Briefly, the tissues were lysed in 300 μL lysis buffer (10 mM Tris, 150 mM NaCl, 1 % Triton X-100, 0.5 % NP-40, 1 mM EDTA, pH 7.4) and mixed with protease and phosphatase inhibitor cocktails (Roche). Nuclear protein was obtained using nuclear and cytoplasmic extraction reagents (Thermo Scientific). Twenty-five micrograms of cell lysate, as quantified with a BCA protein assay (Pierce), was separated on SDS-PAGE and transferred to PVDF membranes (Roche). The primary antibodies used were as follows: anti-glial fibrillary acidic protein (GFAP) (1:4000, Cell Signaling Technology), anti-Iba1 (1:1000, Abcam), anti-DRD2 (1:2000, Abcam), anti-p-STAT3 (Tyr705) (1:1000, Cell Signaling Technology), anti-STAT3 (1:1000, Cell Signaling Technology), anti-CRYAB (1:2000, Abcam), anti-β-actin (1:2000, Abcam), and anti-histone 3 (1:2000, Cell Signaling Technology). The secondary HRP-labeled antibodies were all from Cell Signaling Technology. Chemical reactions were detected with an ECL system (Advansta), and the scanned images were analyzed with ImageJ software (version 1.47). The protocols for cell culture experiments were the same as those described above.

### Quantitative PCR (qPCR)

The messenger RNA (mRNA) levels of inflammatory cytokines were assessed with qPCR. The ischemic penumbra tissues and cultured cells were homogenized on ice, and the total RNA was extracted with Trizol (Sigma). Complementary DNA (cDNA) was synthesized from 1 μg total RNA with an iScript cDNA synthesis Kit (Takara, Tokyo, Japan). Quantitative real-time PCR was performed using a SYBR Premix Ex Taq kit (Takara, Tokyo, Japan). The cycle threshold (Ct) values for IL-1β, IL-6, IL-18, and TNF-α were determined in duplicate and normalized to the endogenous control GAPDH. The qPCR primers were designed using the Primer Picking Program, and their sequences were as follows: IL-1β, forward, 5′-TGCAGCTGGAGAGTGTGGATCCC-3′, reverse, 5′-TGTGCTCTGCTTGTGAGGTGCTG-3′; IL-6, forward, 5′-GGTGCCCTGCCAGTATTCTC-3′, reverse, 5′-GGCTCCCAACACAGGATGA-3′; TNF-α, forward, 5′-ACTTCGGGGTGATCGGTCCCC-3′, reverse, 5′-TGGTTTGCTACGACGTGGGCTAC-3′; IL-18, forward, 5′-GAAGATCTATCACGTAGCCAAGA-3′, reverse, 5′-GCTCTAGACATTCATTAAGGGTTA-3′.

### Co-immunoprecipitation (co-IP) assay

The co-IP procedures were performed as previously described [30]. Protein A/G PLUS-Agarose was purchased from Santa Cruz Technology. The nuclear protein extracts were precipitated with a STAT3 antibody and then analyzed by Western blot with STAT3, p-STAT3, and CRYAB antibodies. STAT3 was used as the loading control in the assay of immunoblot.

### Electrophoretic mobility shift assay (EMSA)

A commercial EMSA Kit (Beyotime Biotechnology) was used to detect the DNA-binding activity of STAT3 following the manufacturer’s instructions. The nuclear protein was extracted as described above. Biotin-labeled STAT3 consensus oligonucleotide probes and cold competitors were used, and the oligonucleotide sequences were: 5′-GAT CCT TCT GGG AAT TCC TAG ATC-3′, 3′-CTA GGA AGA CCC TTA AGG ATC TAG-5′. A total of 20 μg nuclear extract proteins were mixed and then incubated with STAT3 probes for 30 min at room temperature. Samples with loading buffer were separated on 6 % non-denaturing gels and transferred to the nitrocellulose membranes. The chemical reaction was detected with an ECL system (Advansta, Menlo Park, CA, USA).

### Statistical analysis

SPSS (version 19.0) software was used for data analysis in this study. Comparisons among multiple groups were assessed using one-way analysis of variance (ANOVA). Student’s *t* test was used to make intergroup comparisons. For neurological studies, two-way ANOVA was performed for pairwise comparisons between different groups and different periods of time. All the results are expressed as mean ± standard error of mean (SEM). A value of *p* < 0.05 was considered to be statistically significant.

## Results

### Sino dose-dependently reduced ischemic infarct volume and neuronal apoptosis in the MCAO mice

**Fig. 1:**
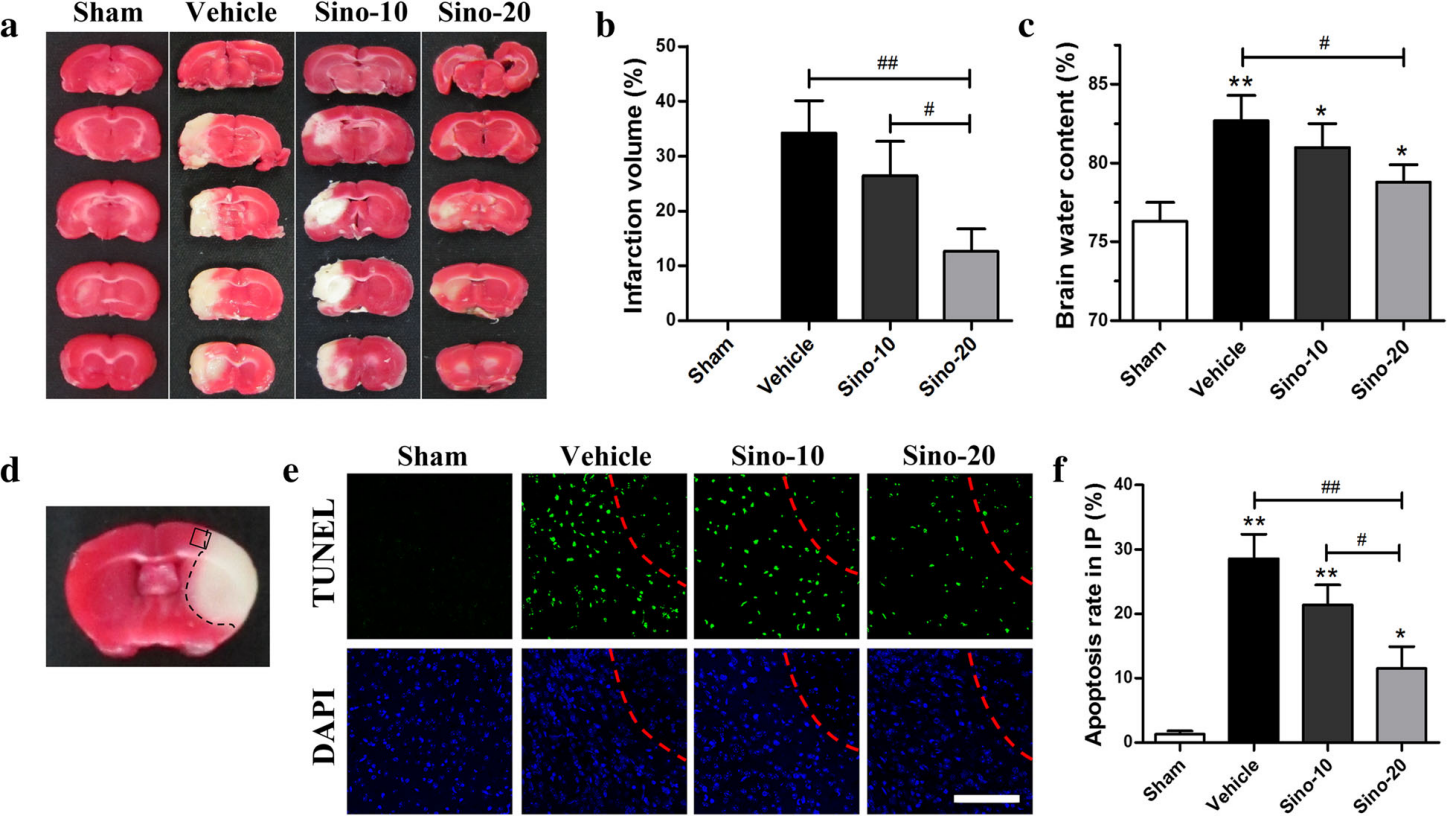
Sino treatment reduced ischemic infarct volume and neuronal apoptosis in the MCAO model. **a** Cerebral infarct volume was assessed via TTC staining 72 h after MCAO. The infarct volume ratio (**b**) and brain water content (**c**) were analyzed in each group. Data are expressed as mean ± SEM (*n* = 5). *p < 0.05 and **p < 0.01 *vs*. sham group; ^#^ *p* < 0.05 and ^##^ *p* < 0.01 *vs*. the indicated groups. The ischemic penumbra area in the box (**d**) was assessed for neuronal apoptosis using TUNEL assay. **e**, **f** Images and quantitative analysis of apoptosis. *Green*, TUNEL-positive cells; *blue*, DAPI. Data are expressed as mean ± SEM (*n* = 5). *Scale bar* = 100 μm. *p < 0.05 and **p < 0.01 *vs*. sham group; ^#^ *p* < 0.05 and ^##^ *p* < 0.01 *vs*. the indicated groups

First, we explored the possible neuroprotective effects of Sino in the MCAO mouse model. Cerebral infarction was assessed by TTC staining 72 h after MCAO. Normal brain tissue was stained *red*, while the infarct lesion remained unstained (*white*). Compared with the vehicle group, the brain infarct volume was decreased in the Sino-10 (10 mg/kg, daily) and Sino-20 (20 mg/kg, daily) groups (Fig. 1a, b). Moreover, the brain infarct volume in the Sino-20 group was less than that in the Sino-10 group (*p* < 0.05). In accordance, the brain water content in the sham group was 76.2 ± 0.43 %. The brain water content in the vehicle group (83.7 ± 0.73 %) was higher than that in the Sino-10 group (81.6 ± 0.69 %) and in the Sino-20 group (77.3 ± 0.54 %) (Fig. 1c). Furthermore, the TUNEL assay was employed to determine the anti-apoptotic effect of Sino in the ischemic mice (Fig. 1e, f). Approximately 193 ± 23 cells stained by DAPI were counted manually in the field per slice. More TUNEL-positive apoptotic cells were observed in the vehicle group than in the sham group (*p* < 0.01). The number of TUNEL-positive cells in the Sino-treated groups was less than that in the vehicle group (*p* < 0.05). In addition, the number of apoptotic cells in the Sino-20 group was markedly less than that in the Sino-10 group (*p* < 0.05). Taken together, the above results indicated that Sino dose-dependently reduced ischemic infarction volume and neuronal apoptosis in the MCAO model.

### Sino alleviated neurological impairment after ischemic injury

**Fig. 2:**
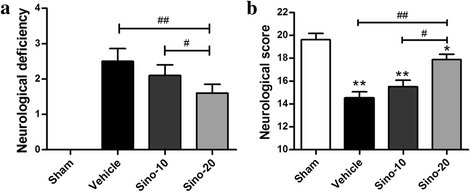
Sino alleviated the neurological impairment of mice after MCAO. The neurological deficiency (**a**) and neurological score (**b**) of mice after MCAO were assessed using a four-tiered grading system and a 21-point scoring system (Garcia test). Data are expressed as mean ± SEM (*n* = 8). *p < 0.05 and **p < 0.01 *vs*. sham group; ^#^ *p* < 0.05 and ^##^ *p* < 0.01 *vs*. the indicated groups

Neurological scoring was conducted to evaluate neurological behavior impairment in different groups (Fig. 2a, b). The mice in the vehicle group exhibited severe neurological deficits compared with the sham group. However, Sino treatment alleviated neurological impairment after ischemic injury, and this protective effect occurred in a dose-dependent manner (*p* < 0.01, vehicle group *vs*. Sino-20 group; *p* < 0.05, Sino-20 group *vs*. Sino-10 group).

### Sino inhibited MCAO-induced IL-1β, IL-6, IL-18, and TNF-α production

**Fig. 3:**
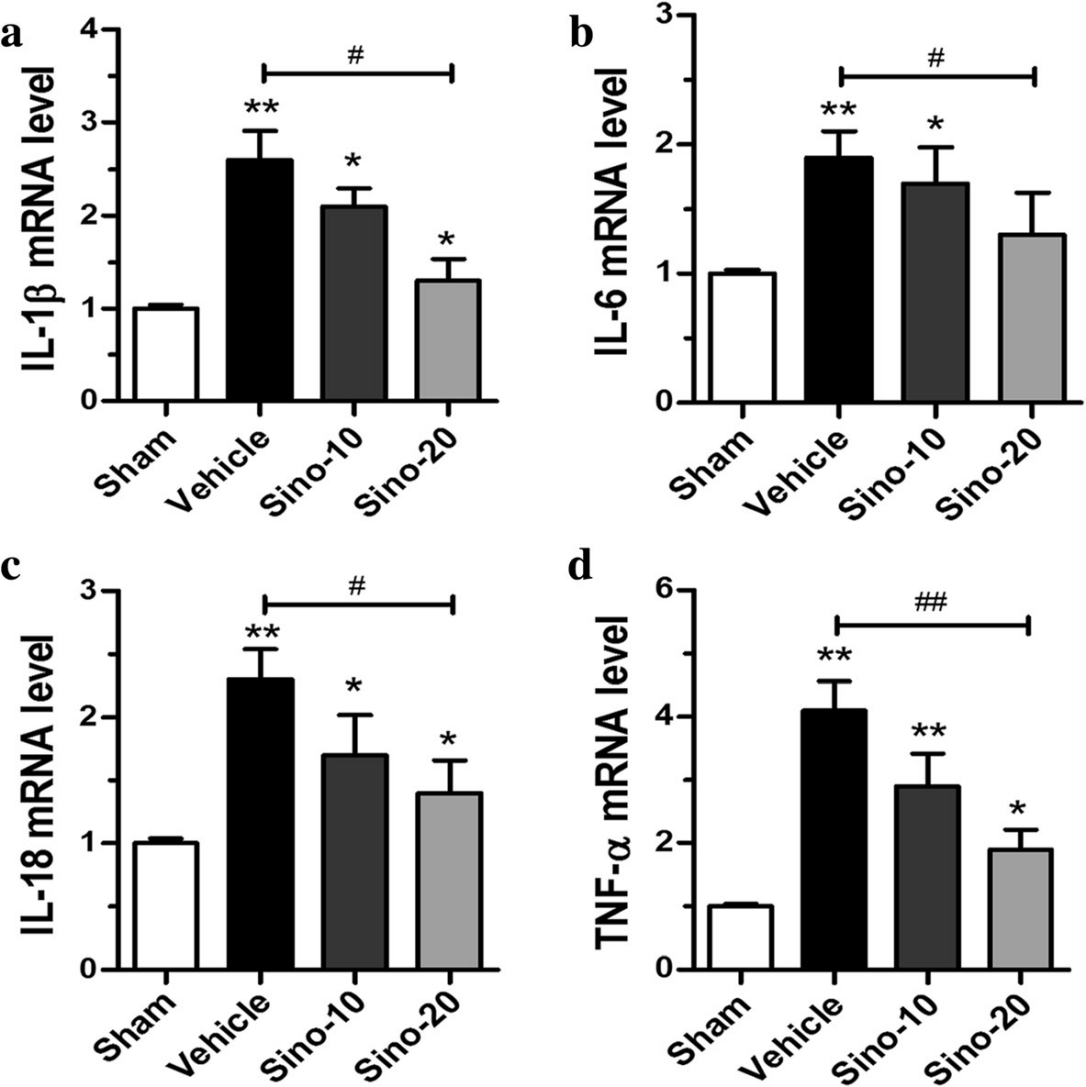
Sino suppressed MCAO-induced neuroinflammation. **a**–**d** qPCR analysis showing the relative mRNA levels of the pro-inflammatory mediators IL-1β, IL-6, IL-18, and TNF-α. Data are expressed as mean ± SEM (*n* = 5). *p < 0.05 and **p < 0.01 *vs*. sham group; ^#^ *p* < 0.05 and ^##^ *p* < 0.01 *vs*. the indicated groups

The IL-1β, IL-6, IL-18, and TNF-α mRNA levels were detected using qPCR. The levels of IL-1β, IL-6, IL-18, and TNF-α in the vehicle group were significantly higher than those in the sham group (*p* < 0.01) (Fig. 3a–d). However, Sino markedly reduced the mRNA levels of the aforementioned cytokines compared with the vehicle treatment in a dose-dependent manner (*p* < 0.05, Sino-10 group *vs*. vehicle group; *p* < 0.01, Sino-20 group *vs*. vehicle group).

### Effects of Sino on astrocytic activation, DRD2, p-STAT3 and CRYAB expression in MCAO mice

**Fig. 4:**
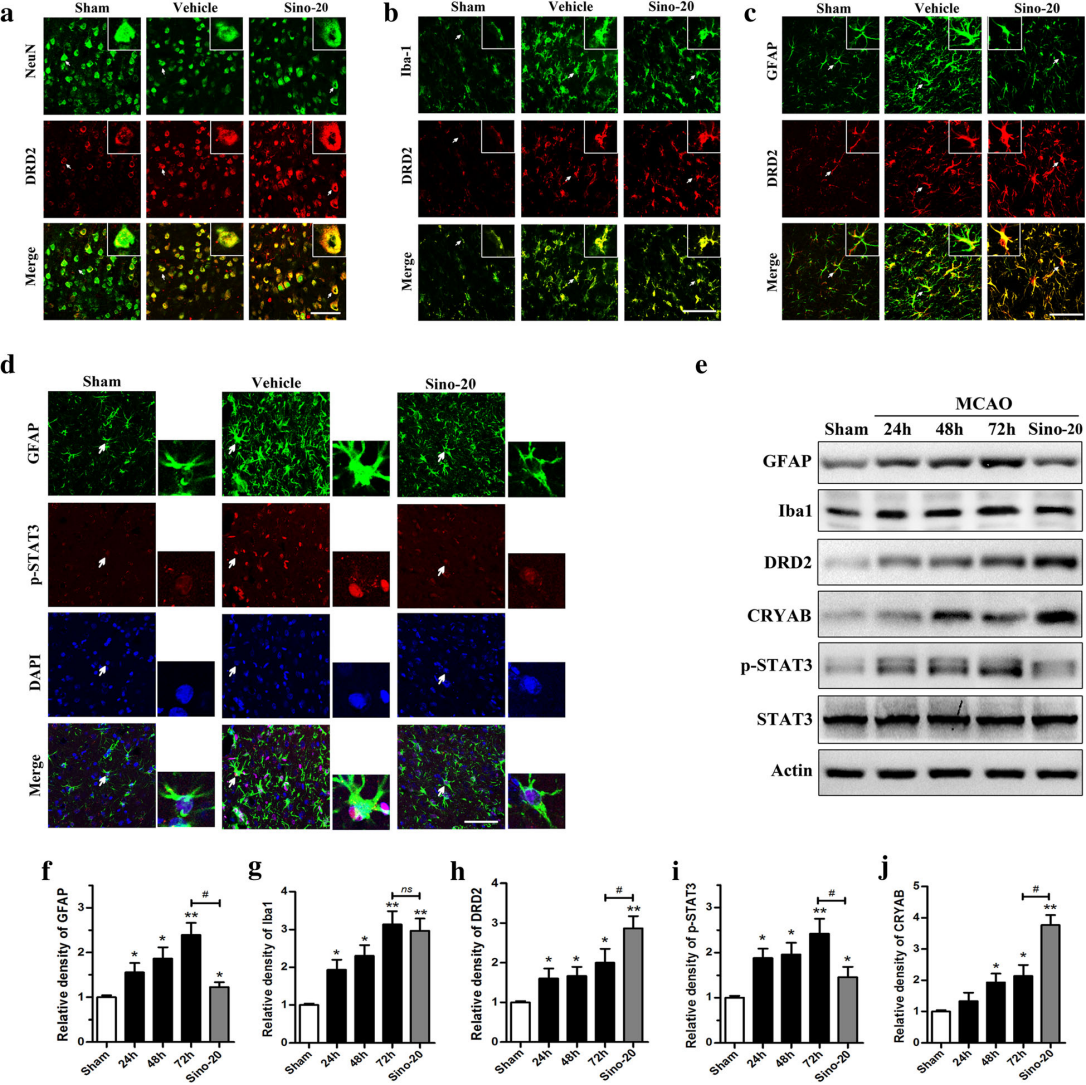
Effects of Sino on the activation of astrocytes and microglia, and the expression of DRD2, p-STAT3, and CRYAB in MCAO mice. **a**–**c** Double-immunofluorescence staining for neuronal (NeuN), microglial (Iba1), and astrocytic (GFAP) DRD2 expression in the ischemic penumbra area after MCAO. **d** Double-immunofluorescence staining for astrocytic p-STAT3 and DAPI. The cells indicated with an *arrow* were magnified. *Scale bar* = 200 μm. **e**–**j** Western blots and quantitative analysis of GFAP, Iba1, DRD2, p-STAT3, and CRYAB expression are expressed as mean ± SEM (*n* = 5). *p < 0.05 and **p < 0.01 *vs*. sham group; ^#^ *p* < 0.05 between the indicated groups

Images from the ipsilateral peri-infarct cortical area (−1.7 to −1.9 mm from bregma) were captured with a laser confocal microscope (Nikon, A1, Tokyo, Japan). Cells were double-labeled with DRD2 combing with NeuN (neuronal marker), GFAP (astrocytic marker), or Iba1 (microglial marker), respectively. The endogenous DRD2 was up-regulated in neurons (NeuN/DRD2, Fig. 4a), microglia (Iba1/DRD2, Fig. 4b), and astrocytes (GFAP/DRD2, Fig. 4c) after MCAO. Sino treatment further increased the DRD2 level in the above cells.

To unravel the potential roles of Sino and DRD2 in the neuroinflammation induced by ischemic injury, we examined the activation status of glial cells (microglia and astrocytes). Astrocytic activation in the ischemic hemisphere was significantly inhibited by Sino treatment (20 mg/kg, daily), as indicated by the lower GFAP level detected with immunofluorescence staining and Western blot (*p* < 0.05, Sino-20 group *vs*. vehicle group) (Fig. 4c–e and Additional file 1: Figure S5). However, neither the changes in Iba1 expression nor the changes in morphology (soma size and ramification index) of Iba1-labeled microglia (Fig. 4b and Additional file 1: Figure S7D-F) differed significantly between the Sino-treated and MCAO groups. A flow cytometry assay separating microglia (CD11b^+^CD45^low^) and macrophages (CD11b^+^CD45^high^) in the ischemic hemisphere after MCAO (Additional file 1: Figure S7B-C) also showed that Sino treatment did not influence the number of activated microglia after MCAO.

In addition, double-immunofluorescence staining also showed that the astrocytic p-STAT3 level was significantly increased after MCAO but was decreased by Sino treatment (Fig. 4d).

The expression of GFAP, DRD2, p-STAT3, and CRYAB were determined using Western blot (Fig. 4e–j). DRD2 was increased as early as 24 h after MCAO compared to the sham group (*p* < 0.05). In addition, Sino further significantly up-regulated DRD2 expression after MCAO compared to the vehicle group (*p* < 0.01). A similar expression pattern was observed in CRYAB. In contrast, Sino significantly reversed the increase in p-STAT3 induced by MCAO.

### DRD2 or CRYAB knockdown abolished the inhibitory effect of Sino on neuroinflammation in OGD-induced astrocytes

The different doses of Sino had no effect on the cell viability (MTT assay) of primary astrocytes (Additional file 1: Figure S1A). The anti-inflammatory effect was observed in the 1 mM and 10 mM Sino groups (*p* < 0.05, *vs*. vehicle group), and there was no significant difference between these two doses (Additional file 1: Figure S1B). Therefore, 1 mM Sino was used in the following in vitro tests.

**Fig. 5:**
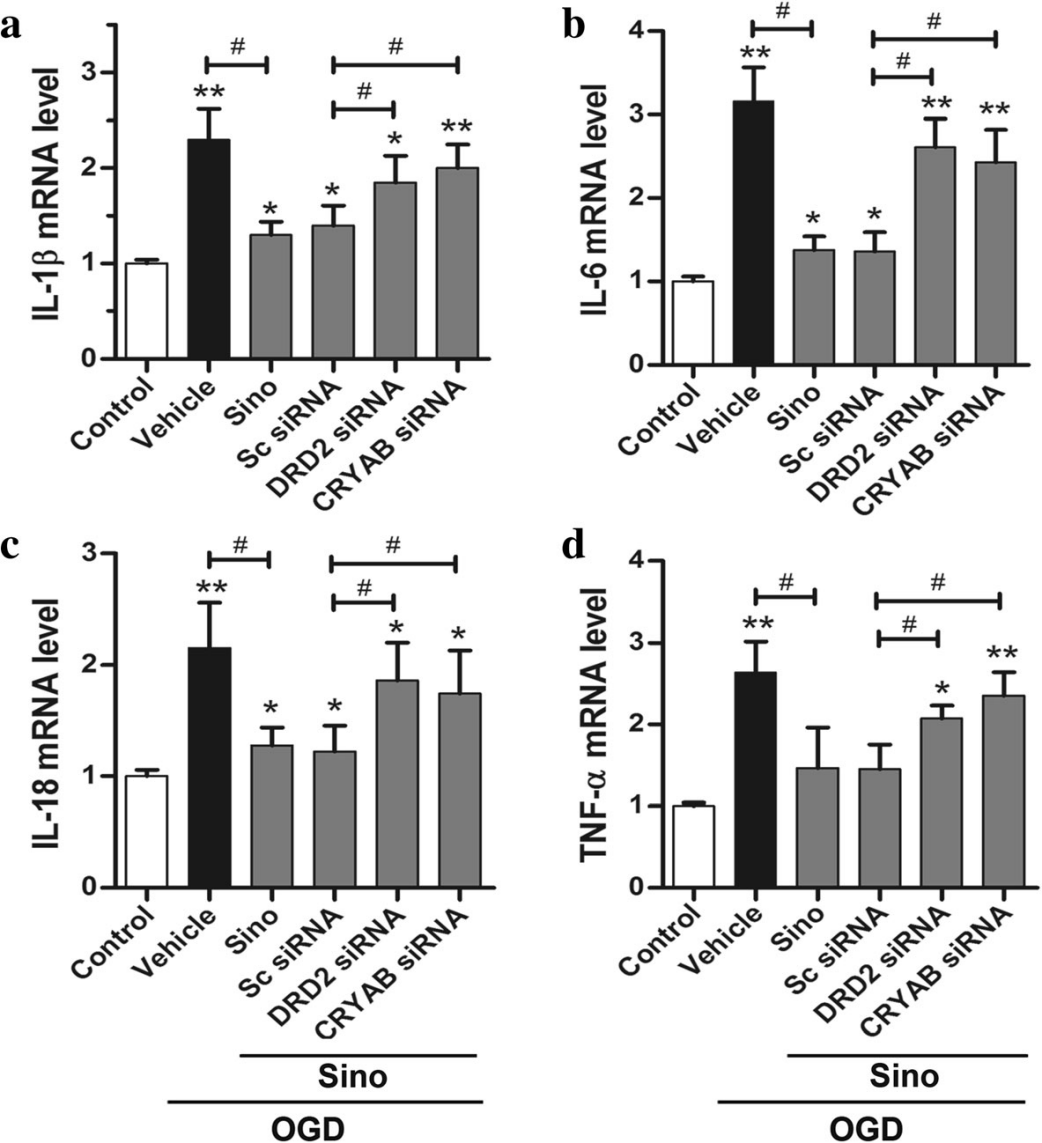
Sino suppressed OGD-induced inflammation in astrocytes in vitro. **a**–**d** qPCR analysis showing the relative mRNA levels of the pro-inflammatory mediators IL-1β, IL-6, IL-18, and TNF-α. Data are expressed as mean ± SEM (*n* = 5). *p < 0.05 and **p < 0.01 *vs*. sham group; ^#^ *p* < 0.05 and ^##^ *p* < 0.01 *vs*. the indicated groups

To clarify the potential role of DRD2 and CRYAB in the anti-inflammatory effect of Sino, we treated primary astrocytes with DRD2 siRNA and CRYAB siRNA. In addition, the cells were maintained under OGD conditions to establish the ischemic in vitro model. Sino treatment significantly reduced the mRNA levels of the pro-inflammatory cytokines IL-1β, IL-6, IL-18, and TNF-α compared to the vehicle group after OGD induction (*p* < 0.05), and this effect was reversed by either DRD2 siRNA or CRYAB siRNA pretreatment (*p* < 0.05, *vs*. Sino + scramble siRNA group) (Fig. 5a–d).

Furthermore, the qPCR (Additional file 1: Figure S2A-B) showed that the DRD2 knockdown astrocytes produced higher mRNA levels of IL-1β than their normal counterparts after OGD induction (*p* < 0.01). In contrast, the expression of the same inflammatory mediator was not significantly altered in the DRD2 knockdown BV2 microglia. In addition, Sino treatment significantly reduced the OGD-induced IL-1β mRNA level in astrocytes (*p* < 0.05), and this effect was significantly blocked by DRD2 knockdown. However, these changes were not significant in microglia in either the DRD2 normal or the knockdown group (Additional file 1: Figure S2A-B).

**Fig. 6:**
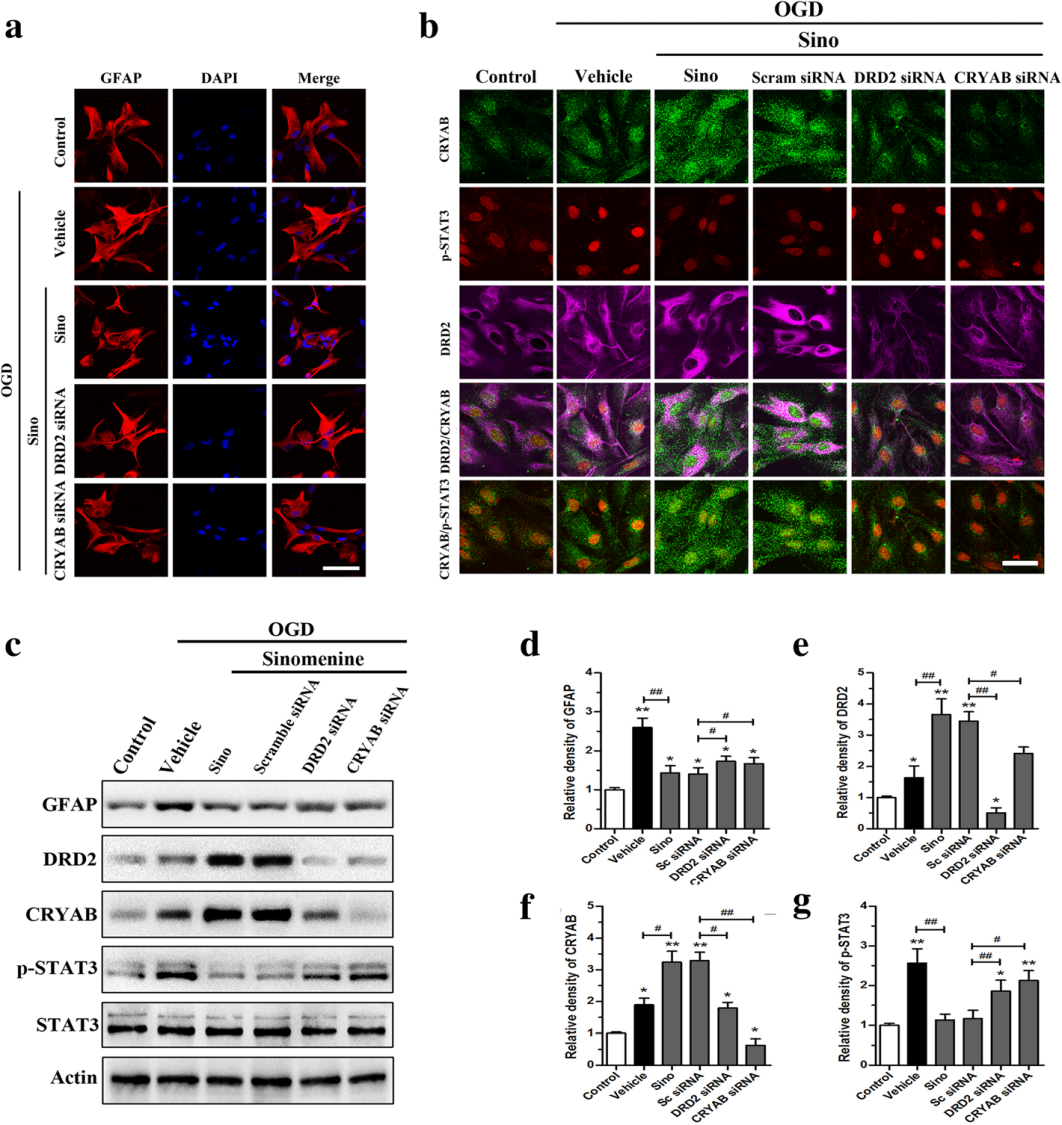
The effects of Sino on the activation of astrocytes and the expression of astrocytic DRD2, CRYAB, and p-STAT3 after OGD induction in vitro. **a** The morphology changes of astrocytes labeled with GFAP. **b** Multi-immunofluorescence staining for CRYAB, DRD2, and p-STAT3 in astrocytes after OGD induction. *Scale bar* = 20 μm (**a**, **b**). **c**–**g** Western blots and quantitative analysis of GFAP, DRD2, p-STAT3, and CRYAB expression. Data are expressed as mean ± SEM (*n* = 5). *p < 0.05 and **p < 0.01 *vs*. sham group; ^#^ *p* < 0.05 and ^##^ *p* < 0.01 *vs*. the indicated groups

Astrocytic morphology changes were detected with GFAP staining (Fig. 6a). OGD-induced astrocytic activation was indicated by increased and lengthened cell protuberances. Sino inhibited the activation of astrocytes after OGD induction, and this effect was weakened by either DRD2 or CRYAB knockdown. To determine the downstream targets of Sino in the anti-inflammatory process, we examined the expression of DRD2, CRYAB, and p-STAT3 in primarily cultured astrocytes. Compared to the control group, OGD significantly increased the levels of GFAP, DRD2, CRYAB, and p-STAT3 in the vehicle group (*p* < 0.05, *p* < 0.01) (Fig. 6b–g). Sino markedly reduced GFAP and p-STAT3 levels and increased DRD2 and CRYAB levels after OGD (*p* < 0.05, *p* < 0.01). These effects were blocked by either DRD2 or CRYAB knockdown, as confirmed with immunofluorescence staining and Western blot (Fig. 6b, c). Interestingly, Sino significantly increased the nuclear expression of CRYAB and inhibited the phosphorylation of STAT3 after OGD in primary astrocytes, with the co-localization of CRYAB and p-STAT3 in the nuclei (Fig. 6b, c). These effects were abolished by either DRD2 siRNA or CRYAB siRNA treatment. There was no difference between the Sino group and the Sino + scramble siRNA group.

### Sino induced the nuclear translocation of CRYAB and attenuated the activation of STAT3 in astrocytes after OGD induction

**Fig. 7:**
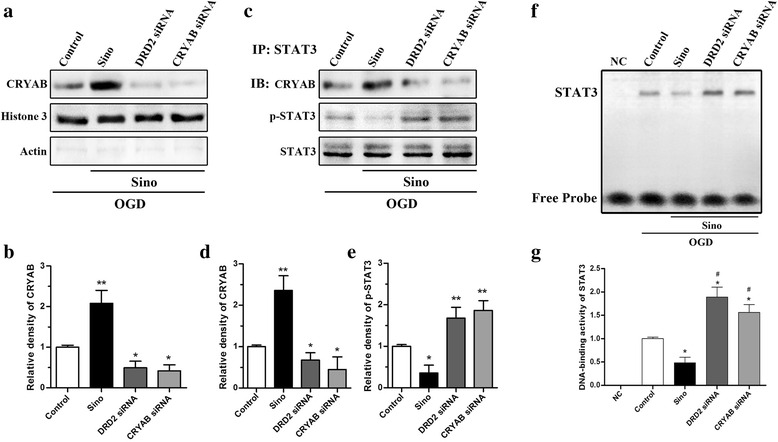
The effects of Sino on the nuclear translocation of CRYAB and the activation of STAT3 in astrocytes in vitro. **a**, **b** Western blot and quantitative analysis of nuclear CRYAB expression. **c**–**e** Immunoprecipitation assay evaluating the binding activity of CRYAB with STAT3 and the phosphorylation of p-STAT3. **f**, **g** EMSA assay of the DNA-binding activity of STAT3. Data are expressed as mean ± SEM (*n* = 5). *p < 0.05 and **p < 0.01 *vs*. sham group

Sino treatment significantly increased the expression of nuclear CRYAB in primary astrocytes after OGD induction (*p* < 0.01, Sino group vs. control group) (Fig. 7a, b). However, DRD2 siRNA or CRYAB siRNA attenuated the nuclear translocation of CRYAB after OGD (*p* < 0.05 *vs*. control group). Co-IP (Fig. 7c–e) showed that Sino enhanced the interaction between CRYAB and STAT3, with increased expression of CRYAB in the immunocomplex after OGD (*p* < 0.01, Sino group *vs*. control group). Additionally, the higher CRYAB levels and the lower p-STAT3 levels were detected in the Sino group. However, these effects were abolished in both the DRD2 siRNA group and the CRYAB siRNA group (*p* < 0.05, *p* < 0.01, *vs*. Sino group). The EMSA assay (Fig. 7f, g) indicated that Sino treatment reduced the DNA-binding activity of STAT3 after OGD induction (*p* < 0.05, Sino group *vs*. control group), which was reversed by either DRD2 knockdown or CRYAB knockdown (*p* < 0.05, *vs*. Sino group).

## Discussion

Ischemic stroke is an important leading cause of morbidity and mortality in adults worldwide, and emerging evidences demonstrate that astrocytic inflammatory responses plays a critical role in secondary brain injury following ischemia [13, 31]. However, the lack of targeted drugs impedes clinical stroke therapy. In the present study, we demonstrated that Sino, a bioactive alkaloid from the Chinese medicinal herb *S. acutum*, significantly attenuated neuroinflammatory injury and neurological deficits in cerebral ischemic mice. Sino ameliorated the activation of astrocytes, the phosphorylation of STAT3, and increased DRD2 and αB-crystallin (CRYAB) expression in vivo and in vitro, all of which were significantly reversed by DRD2 and CRYAB knockdown. We further found that Sino induced the up-regulation and nuclear translocation of CRYAB in astrocytes, and inhibited STAT3 activation by enhancing the interaction between CRYAB and STAT3. Taken together, our findings suggest that Sino exerts potent therapeutic effects in cerebral ischemia by targeting astrocytic DRD2 and suppressing neuroinflammatory injury via the CRYAB/STAT3 pathway.

Neuroinflammation induced by astrocytes is one of the primary causes of secondary brain injury [32]. However, there is a lack of effective therapeutics targeting astrocytes in the clinical treatment. Sino has been successfully used for rheumatoid arthritis treatment for centuries in China [33]. Studies have shown that Sino reduced the LPS-induced synthesis of prostaglandin E3, leukotriene C, TNF-α, and nitric oxide in macrophages and inhibited the proliferation of synovial fibroblasts and lymphocytes stimulated with IL-1β. Sino suppressed ischemia/reperfusion-induced inflammatory infiltration and the expression of CXCL-10, ICAM-1, and TNF-α/IL-6 in kidney cells. In our study, we demonstrated that Sino (20 mg/kg daily for 3 days) significantly alleviated the neuronal damage by reducing cerebral infarction, brain water content, and neuronal apoptosis in the ischemic hemisphere. We also observed the amelioration of neurological deficits of the MCAO mice in the Sino-treated groups. Our findings provided the first evidence that Sino attenuates ischemic brain injury.

It was reported that Sino might exert protective effects by inhibiting acid-sensing ion channel (ASIC) 1a, LDH, and inflammatory factors in neurons and glia [34]. The present study showed that Sino significantly decreased the mRNA levels of pro-inflammatory cytokines, including IL-1β, IL-6, IL-18, and TNF-α, in the tissues surrounding the cerebral infarction (Fig. 3). However, the specific targets of Sino in this anti-inflammatory effect remain undetermined, and the molecular mechanisms of its actions are largely unknown. Astrocytic activation was detected in the penumbra after cerebral ischemia in patients and experimental animal models [9, 35]. Recent studies have demonstrated that astrocytic DRD2 modulates neuroinflammation in the CNS, which suggested a new strategy of suppressing the innate immune response during aging and disease [14, 36]. In our study, we observed significant astrocytic activation and DRD2 up-regulation in the ischemic penumbra area after MCAO (Fig. 4c). Sino remarkably inhibited the activation of astrocytes, rather than microglia, and significantly increased DRD2 expression in astrocytes. However, the activation of microglia after MCAO was not significantly influenced by Sino treatment (Fig. 4b and Additional file 1: Figure S7). We also demonstrated astrocytes were more hyper-responsive in OGD-induced inflammation than microglia when DRD2 was down-regulated. Our findings provided evidence that astrocytic DRD2 mainly contributed to the anti-inflammatory effect of Sino after cerebral ischemia. To identify the downstream effectors of Sino in activating DRD2 signaling in astrocytes, we detected the levels of the endogenous anti-inflammatory protein CRYAB. We found that Sino increased CRYAB expression both in vivo and in vitro (Figs. 4e and 6c). In addition, we also demonstrated that Sino increased nuclear expression of CRYAB in astrocytes, which suggested CRYAB might possess effects of nuclear translocation and transcriptional regulation. However, the mechanism underlying the negative effects of CRYAB on the inflammatory response is not well understood.

The STAT3 signaling has been reported to play a critical role in inflammatory responses in the CNS [37]. STAT3 is activated by phosphorylation at Tyr705, which induces STAT3 dimerization, nuclear translocation, and DNA binding, resulting in the transcription of genes encoding several inflammatory factors [37–40]. In our study, we observed that astrocytic p-STAT3 was up-regulated after MCAO in vivo and after OGD in vitro, and this effect was reversed by Sino treatment (Figs. 4 and 6). In addition, both DRD2 knockdown and CRYAB knockdown increased the p-STAT3 expression. These data suggested that the activation of STAT3 might be involved in the neuroinflammatory injury after MCAO and the neuroprotective effect of Sino could be mediated by DRD2/CRYAB signaling.

In addition, we found that Sino not only increased the expression of CRYAB but also induced the nuclear translocation of CRYAB in vitro after OGD induction (Figs. 6a and 7a and Additional file 1: Figure S3). Additionally, a higher level of nuclear CRYAB was associated with lower level of STAT3 activation. The co-IP showed that Sino enhanced the interaction between CRYAB and STAT3, with a decrease in the level of p-STAT3 in the immunocomplex. Such effects were abolished in both the DRD2 siRNA group and the CRYAB siRNA group (Fig. 7b). The EMSA assay (Fig. 7f) showed that Sino treatment reduced the DNA-binding activity of STAT3 after OGD induction, which was reversed by either DRD2 knockdown or CRYAB knockdown in primary astrocytes. Thus, we inferred that Sino suppressed neuroinflammation by inhibiting the phosphorylation of STAT3 and blocking the DNA-binding activity of STAT3 in astrocytes via DRD2/CRYAB signaling. However, microglial or neuronal DRD2 mechanisms cannot be completely excluded, as some potential indirect neuroprotective effects of Sino could still exist.

## Conclusions

**Fig. 8:**
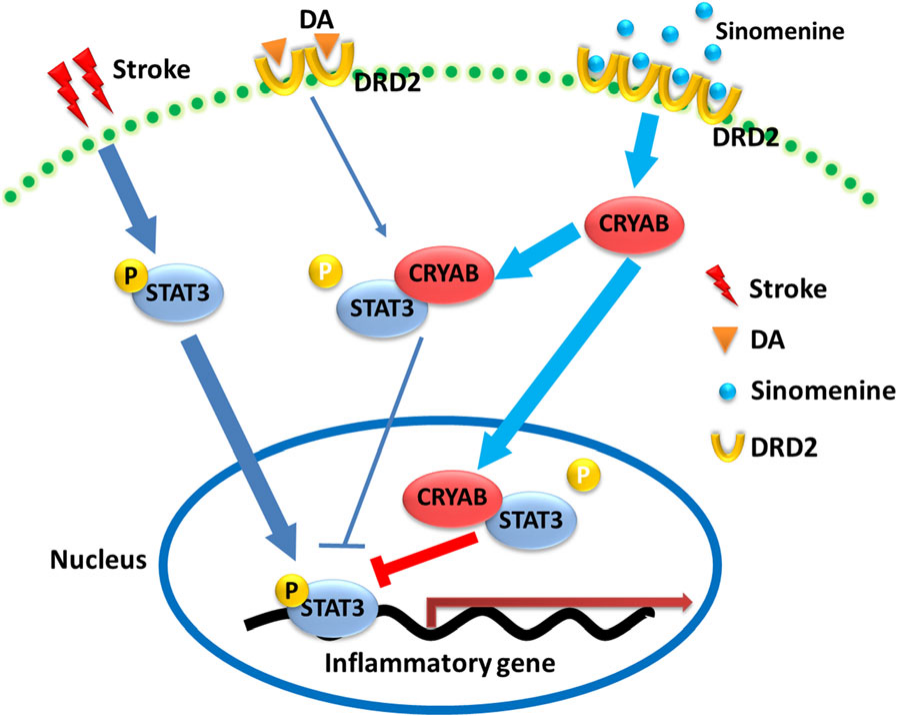
Schematic diagram illustration of Sino suppressing neuroinflammation via astrocytic mechanisms after cerebral ischemic stroke. Briefly, in ischemic stroke, Sino activates astrocytic DRD2 and induces the up-regulation and nuclear translocation of CRYAB. Then, the enhanced interaction between CRYAB and STAT3 blocks the activation and DNA-binding activity of STAT3, which then reduces the generation of pro-inflammatory cytokines and finally contributes to the anti-inflammatory effect of Sino after cerebral ischemic stroke

In summary, our study demonstrates that Sino possesses potent therapeutic effects in cerebral ischemia and suppresses neuroinflammatory injury via targeting astrocytic DRD2 and the CRYAB/STAT3 pathway. Our findings also provide evidence for the clinical use of Sino in ischemic stroke treatment. The schematic diagram in Fig. 8 better illustrates our novel findings in the present study.

## Additional file

Additional file 1: Figure S1. The effect of sinomenine on cell viability and inflammation response in astrocytes. **Figure S2.** Astrocytes were more hyper-responsive in sinomenine suppressing OGD-induced inflammation than microglia. **Figure S3.** Sinomenine increased nuclear expression of CRYAB in astrocytes. **Figure S4.** Blood-brain barrier (BBB) disruption evaluation by Evans blue (EB) extravasation in sham and MCAO mice. **Figure S5.** Effect of sinomenine on the number and activation of astrocytes after MCAO. **Figure S6.** Immunological identification of primary astrocyte culture. **Figure S7.** Effect of sinomenine on the number and activation of microglia after MCAO. Flow cytometry. (DOCX 0.99 MB)

## Abbreviations

cDNA: Complementary DNA
CNS: Central nervous system
CRYAB: αB-Crystallin
Ct: Cycle threshold
DRD2: Dopamine D2 receptor
EMSA: Electrophoretic mobility shift assay
FBS: Fetal bovine serum
GFAP: Glial fibrillary acidic protein
IL-1β: Interleukin-1β
IR: Ischemia and reperfusion
LPS: lipopolysaccharide
MCAO: Middle cerebral artery occlusion
MPTP: 1-Methyl-4-phenyl-1,2,3,6-tetrahydropyridine
OGD: Oxygen glucose deprivation
RA: Rheumatoid arthritis
siRNA: Small interfering RNA
STAT3: Signal transducer and activator of transcription 3
TNF-α: Tumor necrosis factor-alpha
TTC: Triphenyltetrazolium chloride
TUNEL: Terminal deoxynucleotidyl transferase-mediated nick-end labeling
