# Supplementary material for: Sinomenine activates astrocytic dopamine D2 receptors and alleviates neuroinflammatory injury via the CRYAB/STAT3 pathway after ischemic stroke in mice
Source: J Neuroinflammation. 2016 Oct 10;13:263. doi: 10.1186/s12974-016-0739-8 (PMC5057372; doi:10.1186/s12974-016-0739-8)
Supplement: Additional file 1: Figure S1. — The effect of sinomenine on cell viability and inflammation response in astrocytes. Figure S2. Astrocytes were more hyper-responsive in sinomenine suppressing OGD-induced inflammation than microglia. Figure S3. Sinomenine increased nuclear expression of CRYAB in astrocytes. Figure S4. Blood-brain barrier (BBB) disruption evaluation by Evans blue (EB) extravasation in sham and MCAO mice. Figure S5. Effect of sinomenine on the number and activation of astrocytes after MCAO. Figure S6. Immunological identification of primary astrocyte culture. Figure S7. Effect of sinomenine on the number and activation of microglia after MCAO. Flow cytometry. (DOCX 0.99 MB) [file 12974_2016_739_MOESM1_ESM.docx]

**Additional file 1: Supplementary Figure**


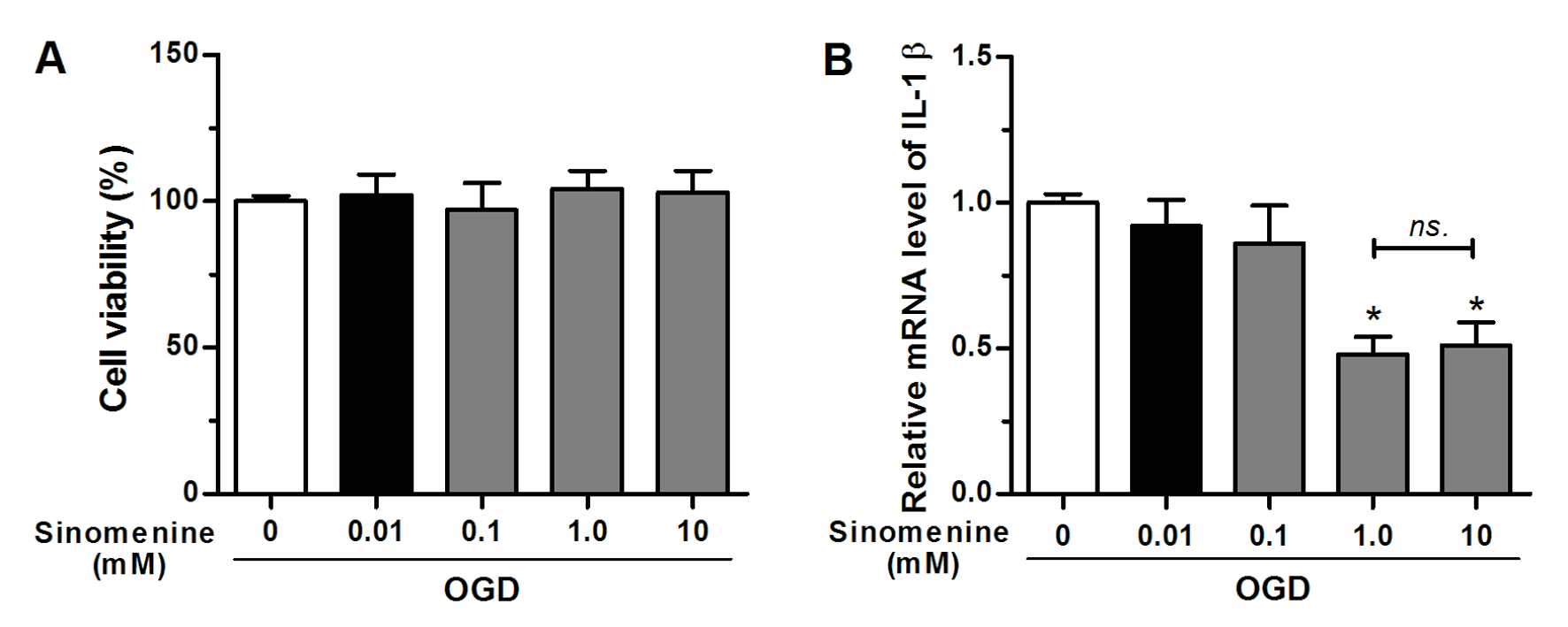


**Figure S1.** The effect of sinomenine on cell viability and inflammation response in astrocytes.

A. MTT assay detecting the cell viability treated with different dosages of sinomenine for 24 h. B. Quantitative PCR analysis of the pro-inflammatory mediator IL-1β mRNA level in astrocytes treated with different dosages of sinomenine for 24 h. Data are expressed as mean ± SEM (n = 6). **P* < 0.05 *vs.* sinomenine (0 mM) group; *ns.*, no significant difference.


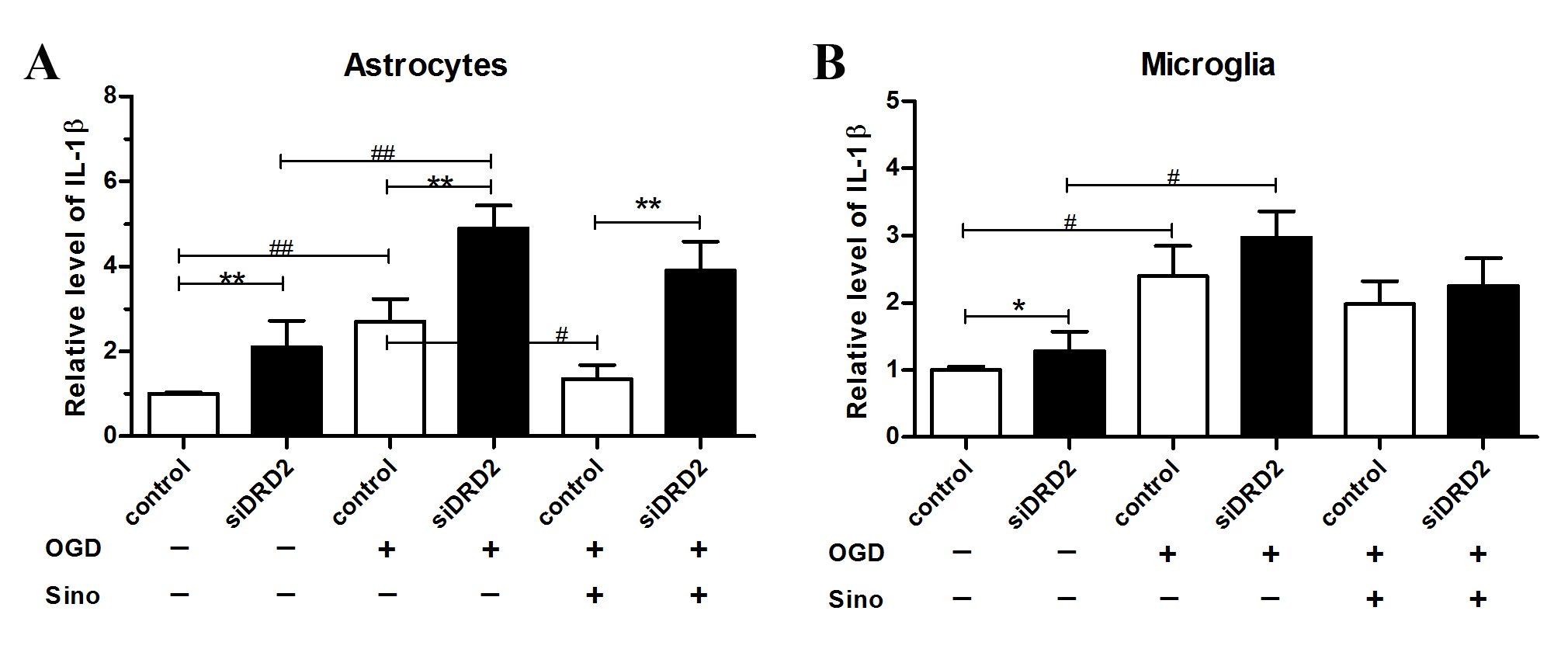


**Figure S2.** Astrocytes were more hyper-responsive in sinomenine suppressing OGD-induced inflammation than microglia.

A-B. Quantitative PCR analysis of the pro-inflammatory mediator IL-1β in astrocyte and microglia after sinomenine treatment (1 mM, 24 h) in control and DRD2 knockdown groups, respectively. Data are expressed as mean ± SEM (n = 6). **P* < 0.05, ***P* < 0.01, ^#^*P* < 0.05 and ^##^*P* < 0.01 *vs.* the indicated groups.


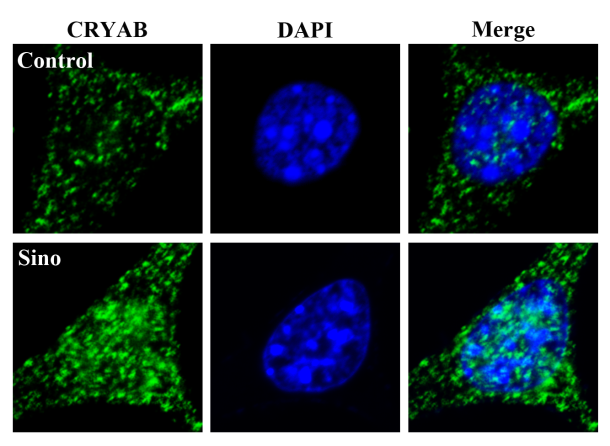


**Figure S3.** Sinomenine increased nuclear expression of CRYAB in astrocytes.

Immunofluorescence detecting CRYAB expression in primary astrocytes after sinomenine treatment (1 mM, 24 h).


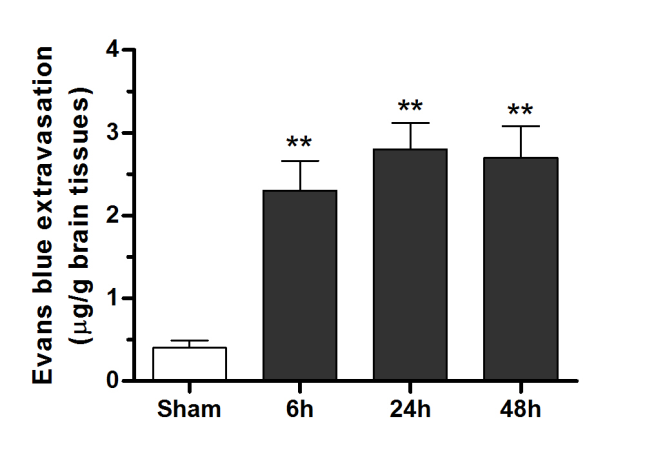


**Figure S4.** Blood-brain-barrier (BBB) disruption evaluation by Evans blue (EB) extravasation in sham and MCAO mice. n = 6 for each groups, P < 0.01, vs. Sham group.


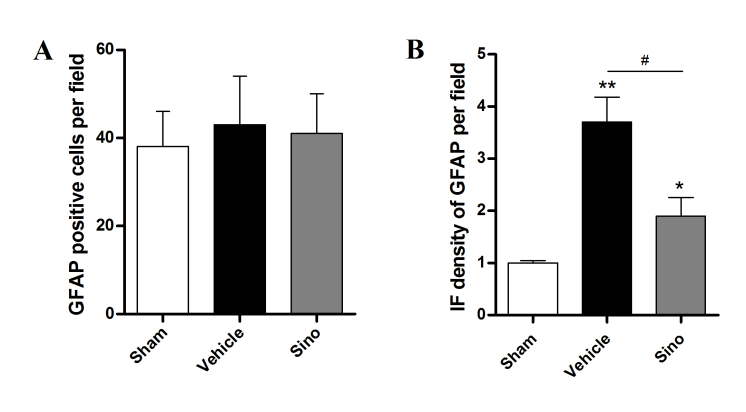


**Figure S5.** Effect of Sinomenine on the number and activation of astrocytes after MCAO. A. The number of astrocytes (GFAP positive cells merge with DAPI) in the field of view per section in Figure 4C and D. B. The immunoreactivity of GFAP in the field of view per section in Figure 4C and D. n = 5 per group, **P < 0.01, *P < 0.05, vs. sham group; #P < 0.05, vs. vehicle group.


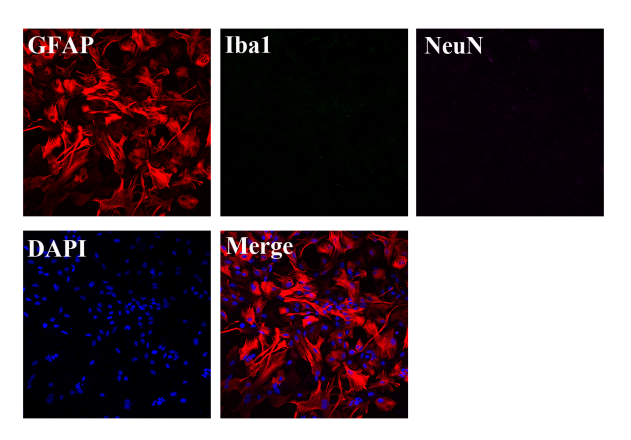


**Figure S6.** Immunological identification of primary astrocyte culture. Astrocytic (mouse anti-GFAP, red), neuronal (rabbit anti-NeuN, purple) and microglial (goat anti-Iba1, green) marker were used for labeling astrocytes, neurons and microglia in the primary astrocyte culture.


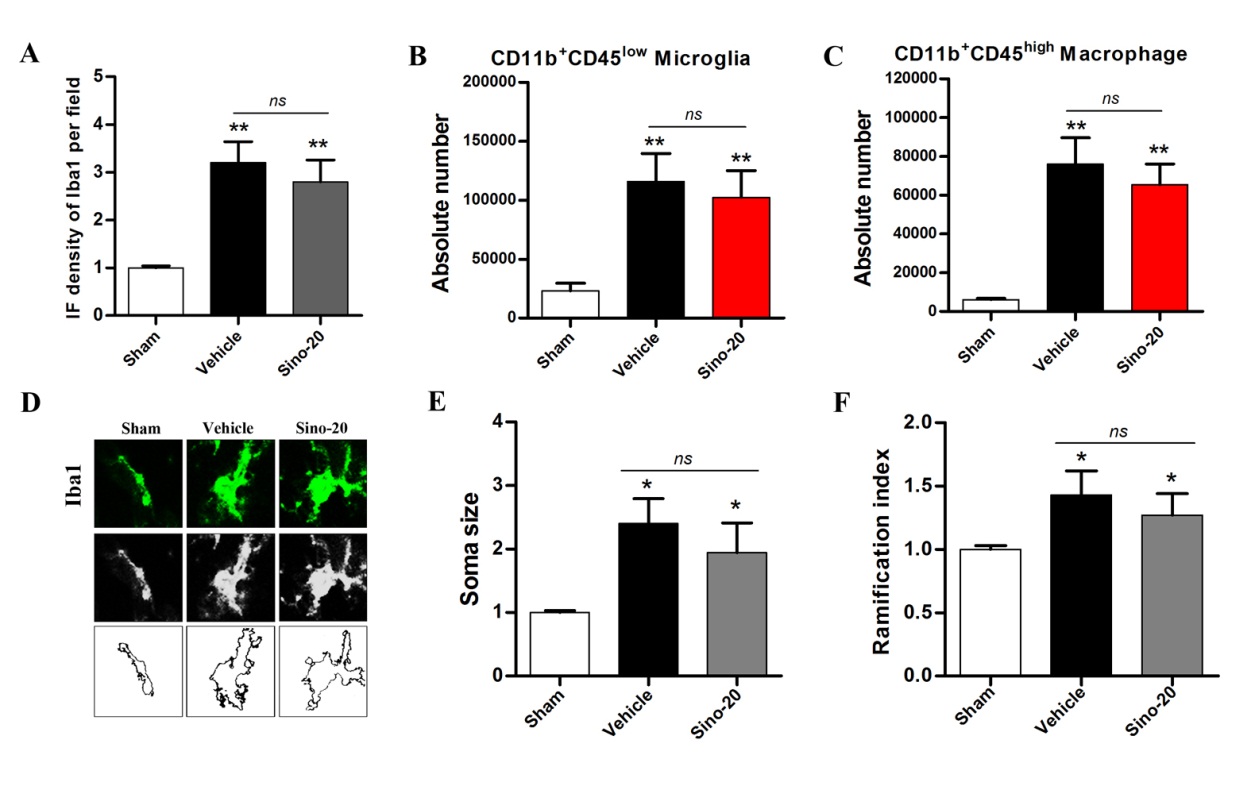


**Figure S7.** Effect of Sinomenine on the number and activation of microglia after MCAO. A. The immunoreactivity of Iba1 in the field of view per section in Figure 4B. B-C. Flow cytometry analysis of absolute number of microglia (marked by CD11b^+^CD45^low^) and macrophages (marked by CD11b^+^CD45^high^) in the ischemic hemisphere after MCAO. D. E-F. Microglial morphology analysis of soma size and ramification index (RI) (calculated as the ratio of cell area/convex area) with Image J software. N = 5 per group, ***P* < 0.01, **P* < 0.05, vs. sham group; *ns*, not significant.

**Flow cytometry**

Anti-mouse antibodies CD11b (BD Pharmingen) and CD45 (Invitrogen) were used for flow cytometry assay. Single cell suspensions obtained from ischemic hemisphere brain tissues after trypsinization were washed with staining medium (PBS containing 0.1% NaN3 and 2% fetal bovine serum). After incubation with mAb, flow cytometry analysis was performed using the Millipore flow cytometer (Guawa 6HT). The data were analyzed using FlowJo software v.7.6.2 (TreeStar, Ashland, OR, USA).
